# Explicit correction of severely non-uniform distributions of cryo-EM views

**DOI:** 10.1107/S2059798326000306

**Published:** 2026-01-21

**Authors:** Charles Barchet, Ottilie von Loeffelholz, Roberto Bahena-Ceron, Bruno P. Klaholz, Alexandre G. Urzhumtsev

**Affiliations:** a Centre for Integrative Biology (CBI), Department of Integrated Structural Biology, IGBMC (Institute of Genetics and of Molecular and Cellular Biology) Centre National de la Recherche Scientifique (CNRS) UMR 7104/Institut National de la Santé de la Recherche Médicale (Inserm) U964/Université de Strasbourg 1 Rue Laurent Fries 67404Illkirch France; b Physics Department Université de Lorraine 54506Vandoeuvre-lès-Nancy France; National Center of Biotechnology, CSIC, Spain

**Keywords:** cryo-EM, 3D reconstruction, view frequency, non-uniform view distribution, preferred orientations, distribution correction, reconstruction improvement, explicit distribution homogenization, image improvement

## Abstract

The quality of three-dimensional macromolecular image reconstruction by cryo electron microscopy (cryo-EM) strongly depends on the number and the quality of the respective two-dimensional projections and on their angular distribution in space. Distributions with one or a few strongly preferred particle orientations may result in maps that are deformed in certain directions. A simple removal of overrepresented views may improve the quality of the reconstructed maps when the level of noise in the two-dimensional (2D) projections is low and the data-set size can afford this removal, but is counterproductive otherwise. Complementarily, giving an increased weight to underrepresented views, or taking multiple copies of them during the reconstruction, may improve the results, naturally, depending on how non-uniform the view distribution is. This work describes the results of three-dimensional (3D) reconstructions using an explicit correction of the number of overrepresented and underrepresented projections for non-uniformly distributed sets. Such correction can be considered as a potential preprocessing, fast and simple, during 3D reconstruction in the image-processing and cryo-EM structure-determination workflow.

## Abstract 2

An explicit numerical leveling of non-uniformly distributed sets of 2D projections with the program *VUE* improves the 3D reconstructions and illustrates sources of image distortion.

## 1. Introduction

The principal object of a single-particle analysis in cryo electron microscopy (cryo-EM) is a three-dimensional map of the electrostatic scattering potential, which is obtained by 3D reconstruction from 2D projections through back-projection (for one of the pioneering publications, see Harauz & van Heel, 1986 ▸). Such maps are the subject of further interpretation, whenever possible, in terms of an atomic model, which has become the norm with the ‘resolution revolution’ (Kühlbrandt, 2014 ▸). A cryo-EM map can contain various kinds of errors. Similar to crystallographic analysis (Lunin *et al.*, 2002 ▸), these errors can be considered either as removable or irremovable. Removable errors are those due to an inappropriate choice of the values for the parameters of the data interpretation; these values can be improved. For example, there are errors in the assigned values of the directions under which given 2D projections correspond to the 3D object reconstructed from them. Exact, or at least better, values of such orientation parameters, for example of the Euler angles (Heymann *et al.*, 2005 ▸), can be obtained during refinement. An example of irremovable errors are errors in the experimental 2D projection values themselves recorded during data collection. Programs can treat some of these (for example inactive pixels on the detector *etc.*), reducing their influence by implementing special protocols, but they cannot be removed.

Missed 2D projections are another example of irremovable errors. It has been known for a while that non-uniformly distributed sets of projections, the views, affect the reconstructed image. Without being exhaustive, we refer to Harauz & van Heel (1986 ▸), Boisset *et al.* (1998 ▸), Unger (2000 ▸), Sorzano *et al.* (2021 ▸), Baldwin *et al.* (2023 ▸) and references therein. The view distributions are analyzed and illustrated with software using different approaches, for example those of Orlov *et al.* (2006 ▸), Punjani *et al.* (2017 ▸), Grant *et al.* (2018 ▸) and the combination of *RELION* (Scheres, 2012 ▸) with *Chimera* (Pettersen *et al.*, 2004 ▸). Measures to characterize the degree of non-uniformity have been proposed (Naydenova & Russo, 2017 ▸; Baldwin & Lyumkis, 2021 ▸; Urzhumtsev, 2026 ▸). An early approach to account for preferential views was a correction based on the point-spread function as implemented in the *IMAGIC* software during 3D reconstruction (Harauz & van Heel, 1986 ▸). Since then, other weighting methods (see, for example, Orlov *et al.*, 2011 ▸; Scheres, 2012 ▸; Sorzano *et al.*, 2021 ▸) have been proposed to correct the respective map distortions.

Recently, we have developed the program *VUE* (Urzhumtseva *et al.*, 2024 ▸), which transforms such distributions of the views into points on a spherical surface and projects them in 2D in a quantitatively exact manner using the Lambert projection, thus avoiding spatial distortions, as opposed to other programs. *VUE* allows the detection and visualization of view distributions as plots, and the calculation of their basic statistics, including the frequency associated with each individual view, ν_*n*_, *n* = 1, …, *N*, according to the number of neighbors and the angular distance from them. Here, we present a further development of this program and a novel tool allowing the correction of non-uniform distributions of views as much as possible, thus improving the respective 3D reconstructions. This method allows an analysis of the effect of such corrections, searching for an optimal protocol. The explicit correction of the list of views requires only a few minutes even for data sets containing of the order of 500 000 two-dimensional projections, making it possible to test several parameter sets and select the optimal updated set either visually or using quantitative measures (Urzhumtsev, 2026 ▸). The 3D reconstruction obtained using this procedure may yield a map that can be used either in addition to, or instead of, the map reconstructed from the original set of projections.

In the following, we explain our approach, describe tests and their results, and provide some examples using simulated and experimental data.

## 2. Methods

### 2.1. Overall scheme

Trying to make the distribution of views more uniform means, first of all, reducing the overrepresented views, as has been performed for decades [for an example, see Shaikh *et al.* (2008 ▸) and references therein]. By doing this, one loses experimental information. Since these views are slightly different and contain different experimental errors, removal of some of them may make the 3D reconstruction noisier and more blurred, *i.e.* decrease its resolution.

On the other hand, one needs to complete the underrepresented views which, formally speaking, requires an additional experiment. In a certain way, this can be performed computationally by introducing respective weighting functions during reconstructions, as mentioned in Section 1.

Instead, one can simulate this explicitly by repeating a reference to a given projection several times, as if it had been recorded several times in exactly the same orientation and containing by chance exactly the same experimental errors. On one hand, this simply repeats the information. On the other hand, this allows the effects of such overweighting of the underrepresented views to be better understood. Moreover, one can artificially modify the parameters of a given projection, its view direction, which could give nonzero estimates for some Fourier coefficients that would otherwise be missed. Both of these modifications are trivial since they do not change the actual file of projections but only the file containing references to them.

### 2.2. Modification of the set of projections

For most cryo-EM software, the values of the 2D projections, available on some regular grid, are kept separately from another file containing the parameters of these projections, usually a line per projection. This list of parameters includes those describing directions (for example using Euler angles) of the projections with respect to the spatial orientation of the object. The simplest procedure to correct non-uniform distribution consists of removing overrepresented views. Practically, in *VUE*, this is performed by removing the respective lines from the list of projection parameters. These lines are selected randomly, with a probability proportional to the excess over the threshold ν_cut_ = *q*_ν_ν_uniform_. Here, ν_uniform_ is the mean frequency corresponding to the uniform distribution and *q*_ν_is the value chosen by the user. Also, the program *VUE* has an option to retain a slight excess of overrepresented views, which may contain extra information, according to Stagg *et al.* (2014 ▸).

A more sophisticated procedure additionally completes the list of projections with extra lines repeating references to the given underrepresented projections. This can be seen as the same projection being ‘accidentally’ measured several times, and the number of copies can be taken to increase its frequency up to the same threshold value ν_cut_, thus equalizing the distribution. These references may be repeated with or without a small artificial random perturbation of the view parameters, *i.e.* the introduction of minor angular variances. In contrast, the projections themselves are not modified.

In this procedure, taking a correction threshold ν_cut_ that is too high leaves more overrepresented experimental projections, and this, to equalize the distribution, requires completion with many underrepresented views, thus introducing too much ‘dummy’ information. Inversely, taking a ν_cut_ that is too low, one reduces the number of ‘dummy’ projections to be added while reducing the number of experimental projections for the reconstruction, which may be counterproductive.

### 2.3. Experimental data

For our analysis, we used experimental data sets available in the laboratory, as well as synthetic data for which the exact answer is known, thus simplifying the evaluation of the results. We identified three experimental data sets that suffered from a non-uniform view distribution.

**Figure 1:**
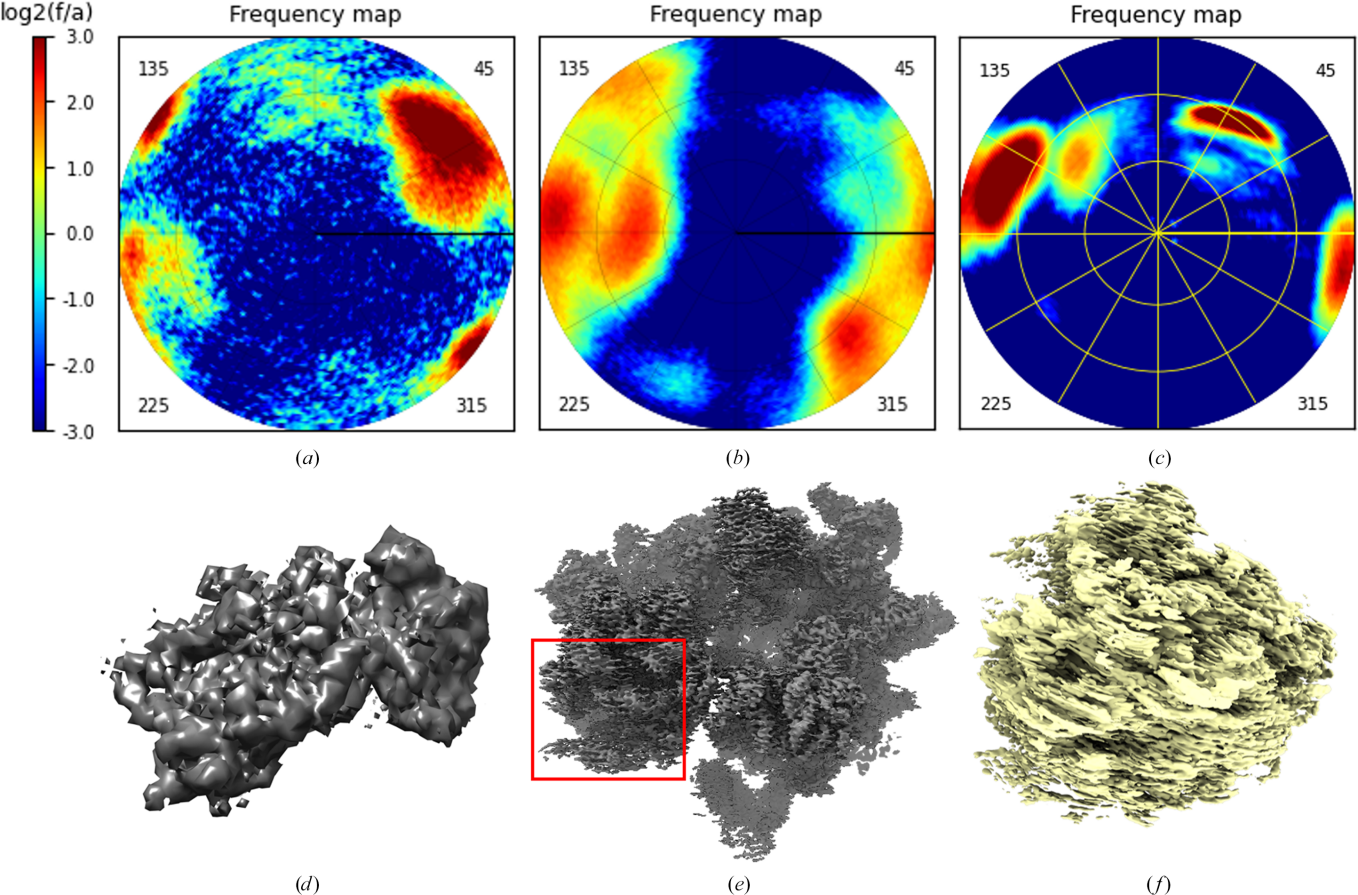
Distributions of views for the experimental data sets used in this work. (*a*) 40S human ribosomal particle; (*b*) 70S *S. aureus* ribosome; (*c*) 80S human ribosome. (*d*), (*e*) and (*f*) show the maps for the respective 3D reconstructions. The color scale is logarithmic (log_2_) with respect to the mean frequency value. The red rectangle in (*e*) indicates the region that is magnified in Fig. 8 ▸(*d*). Maps were plotted with *Chimera* (Pettersen *et al.*, 2004 ▸).

Firstly, we considered the cryo-EM data for a human 40S small ribosomal subunit acquired on the onsite Titan Krios G1 cryo electron microscope installed at the CBI. The data set contains about 45 000 2D projections in total. It has a dominating cluster of projections together with a much smaller one at an angular distance about 90° (Fig. 1 ▸*a*).

Secondly, we worked with about 582 000 2D cryo-EM projections for the 70S *Staphylococcus aureus* ribosome. These data were collected on the in-house Titan Krios G1 cryo electron microscope equipped with a Gatan K2 camera and operating at an acceleration voltage of 300 kV. Fig. 1 ▸(*b*) shows that the views are strongly clustered around three directions distanced by about 45° from each other.

As the third set, we used a cryo-EM data set of the human 80S ribosome, the structure of which had been determined previously (Khatter *et al.*, 2015 ▸; Natchiar *et al.*, 2017 ▸; Holvec *et al.*, 2024 ▸). The data set was acquired on the in-house Titan Krios G4 cryo electron microscope. The 2D projections, about 337 000 in total, are distributed non-uniformly, with four view clusters, of different sizes, concentrated roughly in a plane (Fig. 1 ▸*c*).

For each of these data sets, we calculated the 3D reconstruction using the software *RELION* (Scheres, 2012 ▸). The map for the small subunit was rather isotropic, with no systematic stripes (Fig. 1 ▸*d*). The maps for the 70S ribosome showed some deformation (following the horizontal direction in Fig. 1 ▸*e*). The most significant map deterioration was observed for the 80S ribosome (Fig. 1 ▸*f*).

While the first data set was not useful to test modifications of the sets of views, the two latter gave examples for subsequent analysis and expected improvement of map deterioration on different scales.

### 2.4. Simulated data

To quantify the effect of various manipulations with the sets of non-uniformly distributed 2D projections, we started from a series of tests with simulated data. To facilitate calculations, the relatively small and highly asymmetric structure of initiation factor IF2 was chosen (Simonetti *et al.*, 2013 ▸). Its atomic model was placed in the center of a *P*1 cubic unit cell with an edge length equal to 200 Å. The program *Chimera* (Pettersen *et al.*, 2004 ▸) was used to generate the respective three-dimensional map at a resolution of 2 Å on a grid with a step equal to 1 Å; we refer to it as the 2ÅC map. Additionally, we calculated two similar maps at resolutions of 5 Å (the 5ÅC map) and 10 Å (the 10ÅC map).

**Figure 2:**
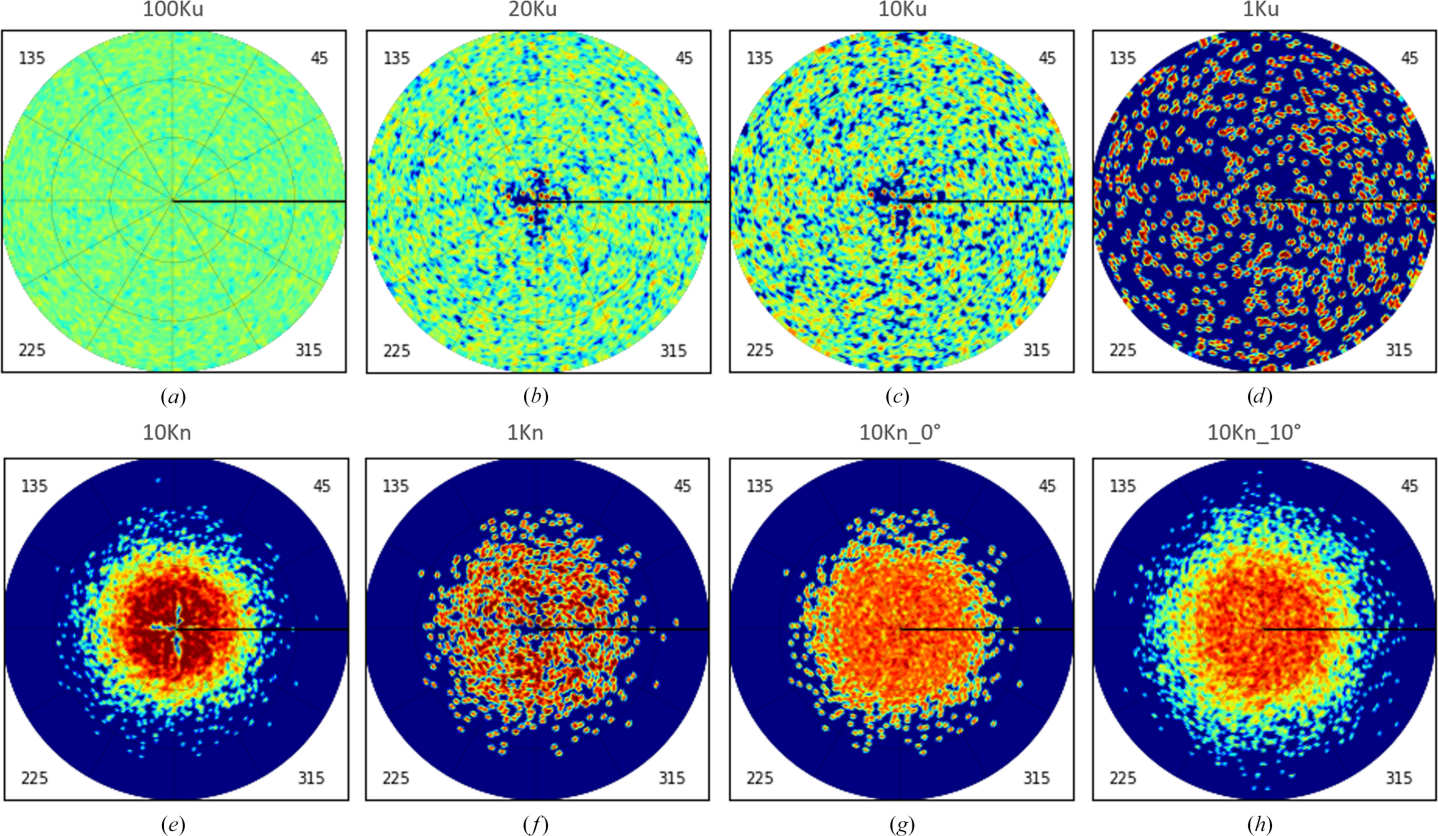
Distribution of views for the simulated data set. (*a*) Initial uniformly distributed set containing 100 000 projections; (*b*) control uniformly chosen selection containing 20 000 projections; (*c*, *d*) uniformly distributed subsets containing 10 000 and 1000 projections, respectively; (*e*) non-uniformly distributed subset 10Kn containing 10 000 projections; (*f*) 1Kn subset (1000 projections) of 10Kn after removing the most overrepresented views; (*g*) 10Kn subset corrected by replacing ∼3600 overrepresented views by ‘dummy’ copies of underrepresented views; (*h*) the same as (*g*) but with perturbation of the orientation of ‘dummy’ copies with a mean of 10°. The color scheme is the same as in Fig. 1 ▸.

The software *RELION* (Scheres, 2012 ▸) was used to generate 100 000 two-dimensional projections of this 3D map, also on a grid with a step size of 1 Å. The directions of these projections were distributed randomly and uniformly in space (Fig. 2 ▸*a*), and we refer to this set as 100Ku.

Using the same atomic model, we calculated Fourier coefficients at a resolution up to 2 Å from the exact distribution of the electrostatic scattering potential (Peng, 1999 ▸) and computed a map using Fourier transform (FT) with these coefficients (the 2ÅFT map).

### 2.5. Tools to analyze map quality

#### 2.5.1. FSC curves

To quantitatively estimate the quality of the 3D reconstructions, we used several measures, including the commonly used FSC curves calculated for two half-maps (Saxton & Baumeister, 1982 ▸; van Heel *et al.*, 1982 ▸), fully understanding that this value (or rather the uncertainty in these values) may be affected by the total number of projections. Such curves show consistency of the Fourier coefficients and therefore the confidence of the map details. At the same time, this does not exactly define the size of the details seen in the map, although it is relevant to it [see, for example, Afonine *et al.* (2018 ▸) and references therein]. For the tests with simulated data, we also calculated the FSC between the reconstructed and the control maps as a measure of the map closeness.

**Figure 3:**
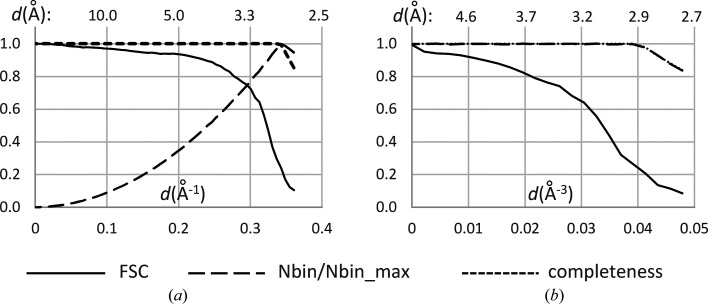
Scaling of FSC curves for the 80S ribosome data. (*a*) FSC curve calculated as a function of resolution on the conventional scale uniform in Å^−1^; the tremendous variation in the number of Fourier coefficients per bin is illustrated by the dashed line. (*b*) The same curve but calculated on a uniform scale in Å^−3^, giving a near-equal number of Fourier coefficients in all resolution shells. The dotted line shows data completeness; in (*b*), it coincides with the dashed line.

In cryo-EM, the FSC curves are traditionally computed and plotted as a function of the resolution expressed in Å^−1^. Calculations on the respective uniform scale lead to a strong statistical imbalance between the number of Fourier coefficients per bin, which varies drastically with the resolution. Fig. 3 ▸(*a*) illustrates this for the 80S ribosomal data set described above. For an equilibrated binning, we introduced a scale uniform in Å^−3^, as previously used in crystallography (Fokine & Urzhumtsev, 2002 ▸; Liebschner *et al.*, 2019 ▸). Bins with bounds uniformly chosen on such a scale contain roughly equal numbers of data (Fig. 3 ▸*b*), which is statistically more appropriate (Urzhumtsev, 2025 ▸). Another advantage of this type of scale is that it increases the sensitivity in the region of high spatial frequencies. In other words, it tends to zoom in on the right-hand part of the curve, which is the high-resolution interval of interest.

#### 2.5.2. Map-discrepancy *D* function

Whatever the resolution scale, the FSC curve is a characteristic of the map Fourier coefficients and not of the map itself. To analyze the map quality, we avoided introducing extra objects such as atomic models. Instead, to characterize how close two maps are to each other, we used the map-discrepancy function, a measure which corresponds to a visual map comparison (Lunin, 1988 ▸; Urzhumtsev *et al.*, 2014 ▸).

Firstly, one chooses the percentile value *p* = *N*_selected_/*N*_grid_of the unit-cell volume, where *N*_grid_ is the total number of grid nodes in each of the two maps. Then, for each couple of maps, we identify the cutoff levels ρ_1_ and ρ_2_ which select the respective number *N*_selected_ of grid nodes with map values above the respective cutoffs. Finally, we analyze the difference between the selected regions by computing the number *N*_diff_ of grid points by which the masks of these regions differ and normalizing the computed value with respect to the theoretical number obtained for comparison with a random-valued map. By varying the percentile *p*, we obtain the discrepancy function *D* defined as
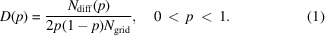
Values of *D*(*p*) close to zero indicate a high map similarity at a given percentile *p*, while values close to one mean that the obtained maps are near-random compared with each other at this particular percentile. The regions of interest, those selected at the 1σ–2σ level in normalized crystallographic maps, usually correspond to *p* values in the limits 0.01–0.05 (Urzhumtsev *et al.*, 2014 ▸). Regions of interest around the macromolecule in cryo-EM maps are an order of magnitude smaller due to much looser molecular packing in the virtual unit cells.

### 2.6. Control 3D reconstruction for simulated data

**Figure 4:**
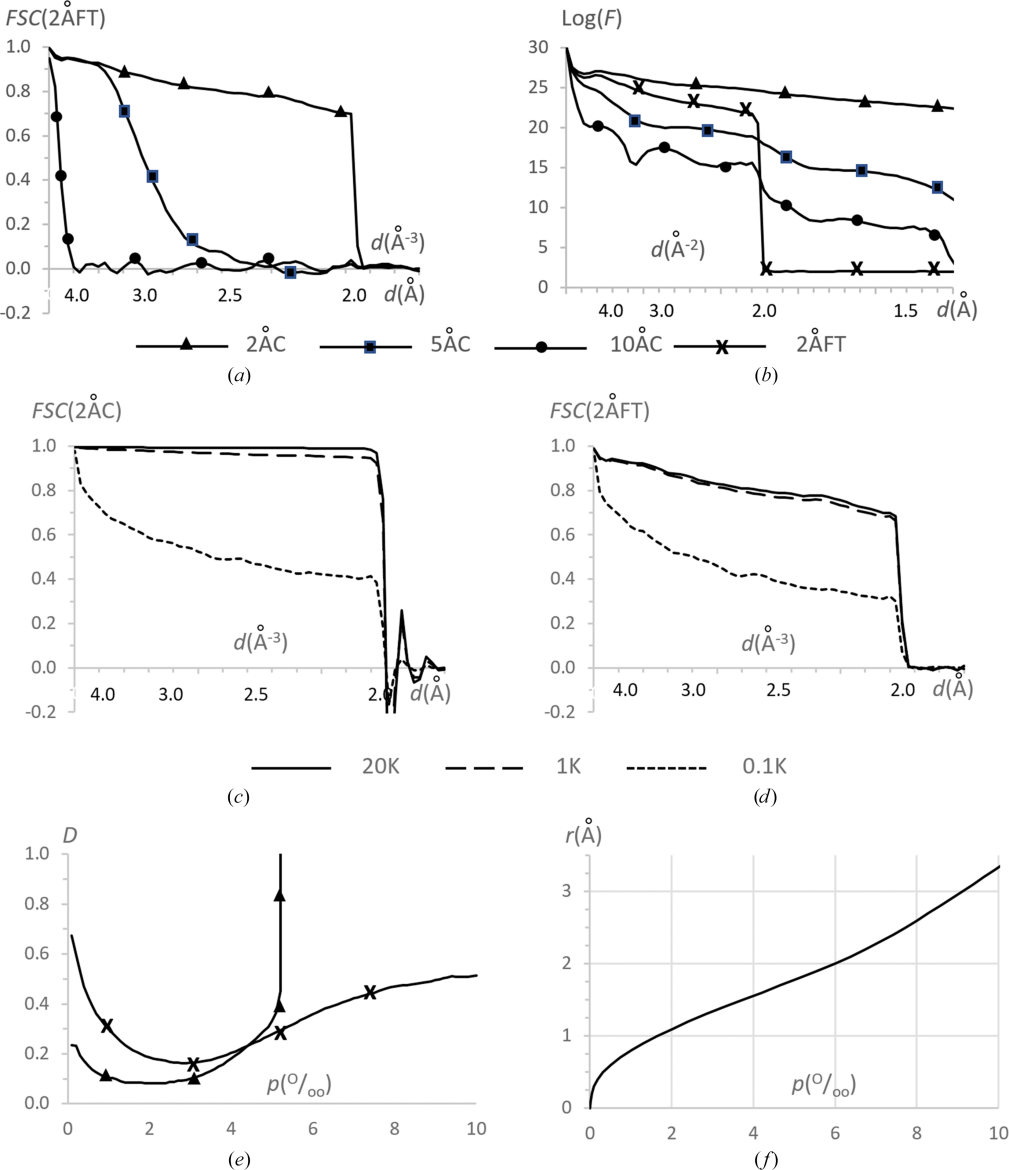
Analysis of the control maps. (*a*) FSC curve between maps of different resolution calculated by *Chimera* and the 2 Å resolution map calculated with FT; the resolution scale is uniform in Å^−3^. (*b*) Logarithm of the mean intensity of the Fourier coefficients as a function of resolution calculated for the same maps as in (*a*); the resolution scale is uniform in Å^−2^. (*c*) FSC curve between maps reconstructed with different numbers of projections selected randomly and uniformly and the 2 Å resolution map calculated by *Chimera*; the resolution scale is uniform in Å^−3^. (*d*) The same comparison with the 2 Å resolution map calculated by FT. (*e*) *D* function between the 20Ku reconstruction and the 2 Å resolution maps calculated by *Chimera* and by FT. (*f*) Atomic radius giving the molecular mask of the required volume, expressed in per thousand in both (*e*) and (*f*).

Before creating the control 3D reconstruction from the simulated 2D projections, we applied the proposed validation tools to the two simulated maps, 2ÅC and 2ÅFT, defined in Section 2.4. As expected, they were different from each other (Fig. 4 ▸*a*). In particular, the FSC between them decreased to 0.7 when the resolution approached 2 Å. A similar effect, with a different sharpness, was observed for two other control maps calculated by *Chimera* and by FT at 5 and at 10 Å resolution (Fig. 4 ▸*a*).

To explain such a difference, we computed the mean intensity of the Fourier coefficients of these maps as a function of the resolution. The map calculated as a Fourier transform showed an expected sharp decrease in the intensity of the Fourier coefficients at 2 Å resolution to the noise level (Fig. 4 ▸*b*). However, there are data at a resolution higher than 2 Å for all three maps calculated by *Chimera* (Fig. 4 ▸*b*). This can be explained if these maps were actually calculated as sums of atomic contributions taken as a ‘peaky’ function approximating the central peak of atomic images at the respective resolution, for example, the Gaussian (see, for example, Diamond, 1971 ▸). In other words, decreasing the resolution was modeled by increasing the *B* factor. The increased slope of the respective curves when decreasing the nominal resolution (Fig. 4 ▸*b*) corroborates with this hypothesis. Such modeling allows the fast computation of an approximate map while ignoring the actual shape of atomic images at a given resolution, including respective Fourier ripples, resulting in certain map distortions (see, for example, Urzhumtsev & Lunin, 2022 ▸).

We then made several 3D reconstructions from the generated 2D projections using the software *RELION* with the 10 Å map as the reference object. As expected, using all 100 000 error-free projections for a small object was excessive. Instead, we calculated reconstructions with 20 000 projections or lower, selecting them randomly and uniformly from this 100Ku set (Figs. 1 ▸*b*–1 ▸*d*). The Fourier coefficients of such reconstructions, performed with several thousand projections, were very close to those for the 2ÅC map (not shown). For sets with a number of projection smaller than approximately 1000 (referred to as the 1Ku map in the following), the coefficients of the reconstructions started to have significant errors. Figs. 4 ▸(*c*) and 4 ▸(*d*) show this for the set composed of 100 projections. As a result of this analysis, we decided to consider the map calculated with 20 000 uniformly selected projections (referred to as 20Ku in the following) as the reference (the best reconstruction expected). Maps composed of a smaller number of 2D projections, from 10 000 to 1000, were used to simulate imperfect reconstructions.

To further analyze the 20Ku map that represents the best 3D reconstruction, we calculated how much it differs from each of the two maps, the initial 2ÅC map and the Fourier-calculated 2ÅFT map. The *D* function shows that the highest valued points of 20Ku, roughly for *p* < 0.003, are close to those in both of these maps while being closer to the 2ÅC map. The points corresponding to *p* > 0.005 are closer to those in the 2ÅFT map (Fig. 4 ▸*e*). For the intermediate values, all three maps are relatively close to each other. If one calculates a model mask as a set of spheres of a given radius *r*_atom_ and centered on atoms, these percentile values of 0.003 and 0.005 correspond roughly to *r*_atom_ = 1.36 Å and to *r*_atom_ = 1.78 Å (Fig. 4 ▸*f*). Below, we refer to the latter mask as the molecular region.

These results agree with the fact that 20Ku reproduces the peaks around atomic centers (accurate in the 2ÅC map), while it ignores the Fourier ripples which are present in the maps of limited resolution (2ÅFT map). The ripples of a given atom modify the map at some distance from the center, while the peak values themselves are perturbed by ripples from the neighboring atoms (Urzhumtsev *et al.*, 2022 ▸). Both of these corrections are not reflected in the 2ÅC map and therefore in the 2D projections used to reconstruct 20Ku.

A visual comparison of these maps at conventional cutoff levels selecting the molecule (varying about 20σ in the given maps) showed practically no difference between the initial 2ÅC map and both the 20Ku and 1Ku 3D reconstructions (not shown).

## 3. Results

### 3.1. Reconstructions with simulated data

#### 3.1.1. Reconstructions with non-uniformly distributed views

**Figure 5:**
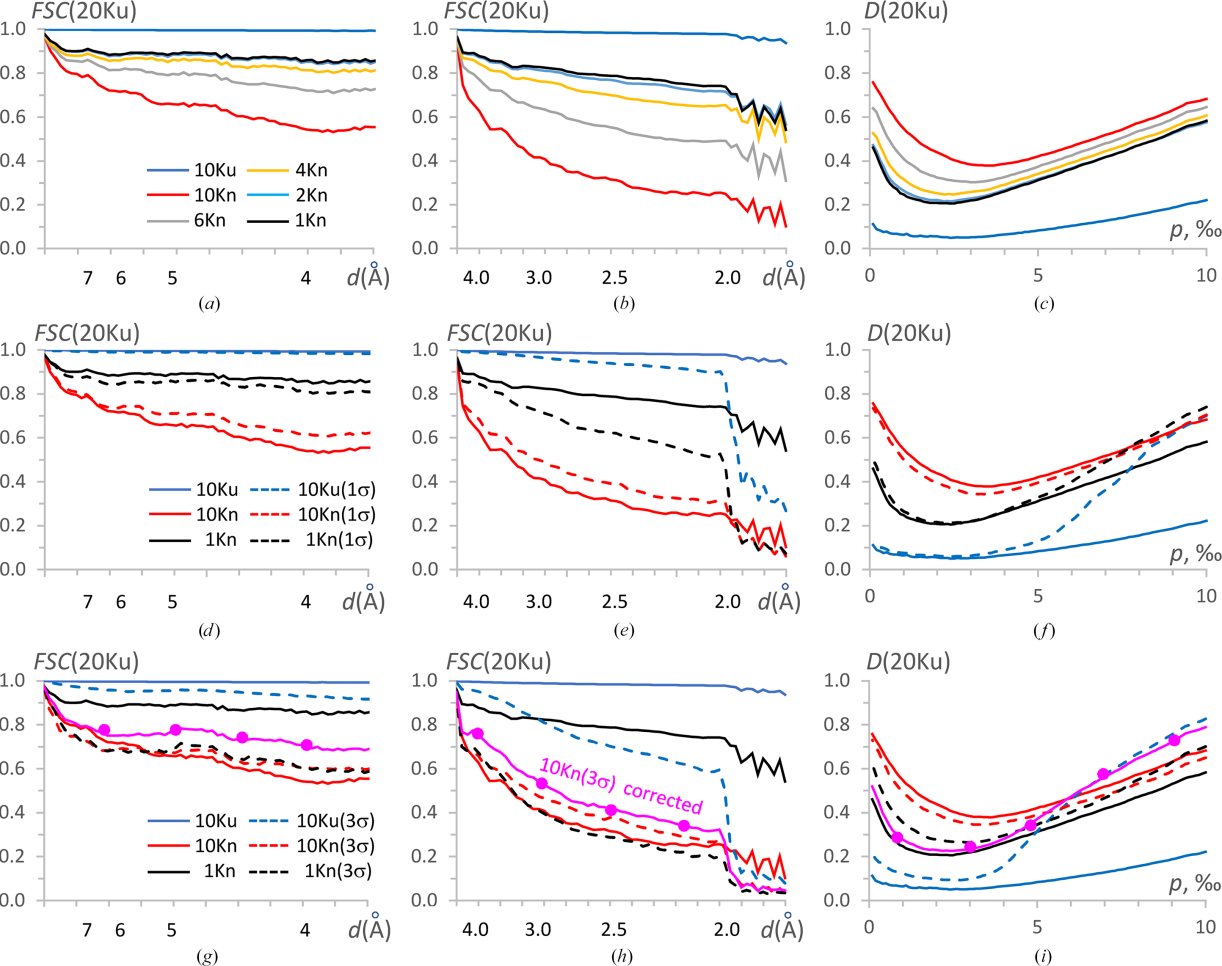
Data analysis for the IF2 simulated data. FSC curves calculated between different maps and the reference map 20Ku in lower (left column) and higher (medium column) resolution intervals; the resolution scale is uniform in Å^−3^, while values in Å are given for reference. The right column shows the respective *D* functions as a function of per thousand unit-cell volume. The upper row shows plots for the 10Ku map for the uniformly distributed 10 000 views (blue), the 10Kn map for the non-uniformly distributed 10 000 views (red) and the maps calculated with the reduced non-uniformly distributed sets of projections up to 1000 views (black). The medium row shows, as full lines, the calculation with the error-free sets 10Kn and 1Kn, and, as dashed lines, the reconstructions with the same sets but with 1 r.m.s.d. errors in the projection values; the color code is the same as above. The bottom row is similar to the medium row but for data with 3 r.m.s.d. errors in the projection values. The curves in magenta, with markers, are for the set of projections with ∼5000 severely overrepresented projections removed and replaced by ∼1000 ‘dummy’ projections for the most underrepresented projections.

To analyze the effect of a non-uniform distribution of views, we generated two subsets of views of the full set at 100 K. The views for the first subset, referred to as 10Ku, were selected uniformly. Reconstruction with this set resulted in a map giving an FSC with the reference map 20Ku equal to one for all resolution shells lower than 2 Å, the imposed resolution for the initial map. The FSC values are actually quite high even for higher resolution Fourier coefficients, which are nonzero for these two maps (blue curves in Figs. 5 ▸*a* and 5 ▸*b*).

**Figure 6:**
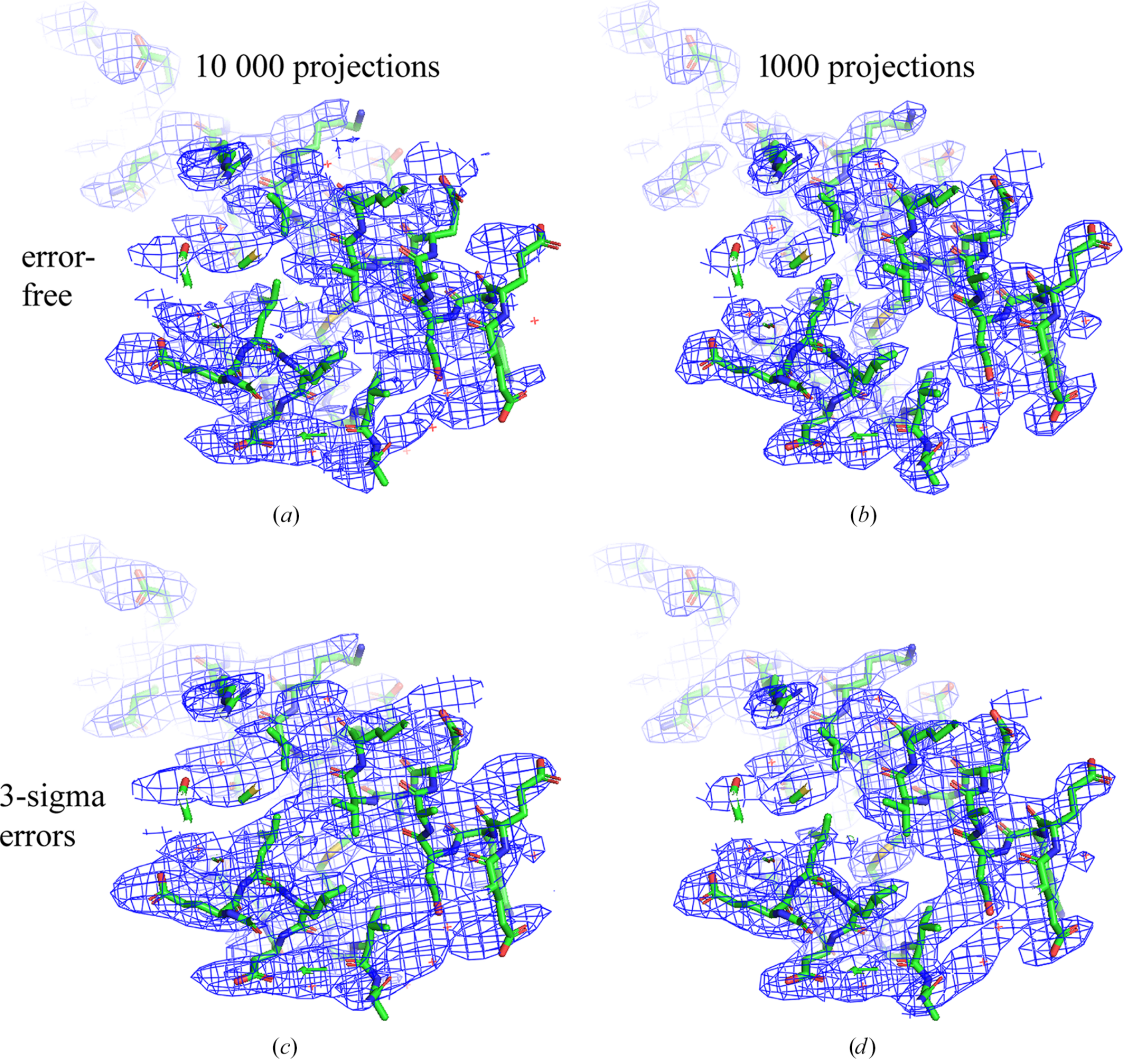
3D reconstructions for the IF2 simulated data. Reconstructions performed with 10 000 non-uniformly distributed projections (left column) and with 1000 left after removing the overrepresented projections (right column). The upper row shows the results of the reconstruction with the error-free 2D projections; the bottom row shows those with 3 r.m.s.d. errors in the projection values. The maps in (*b*) and (*d*) are less stripy in comparison with those in (*a*) and (*c*), respectively, while (*d*) shows some residual deformation in comparison with (*b*). This figure was prepared with *PyMOL* (Schrödinger).

The second subset, referred to as 10Kn, contained the same number of 2D projections but concentrated around one axis (*i.e.* a preferential view), chosen for simplicity as *Oz*. These views were selected from the 100 K set according to the two-dimensional normal distribution. Figs. 2 ▸(*c*) and 2 ▸(*e*) illustrate these subsets 10Ku and 10Kn. For the reconstruction with the 10Kn set, the FSC drastically falls, decreasing near-linearly on the inverse cubic resolution scale (Å^−3^) both for higher and lower resolution shells (red curves in Figs. 5 ▸*a* and 5 ▸*b*). The map for the respective 3D reconstruction appears to be elongated in one direction (Fig. 6 ▸*a*), consistent with the existence of preferential particle orientations.

The principal goal of the following tests was to improve, as much as possible, the latter 3D reconstruction by correcting the 10Kn set with no additional projections taken at new angles.

#### 3.1.2. Reducing overrepresented views, error-free projections

As a first approach, we equalized, as much as possible, the distribution of views in the 10Kn set by removing the most overrepresented views, step by step, using the program *VUE*. Figs. 5 ▸(*a*) and 5 ▸(*b*) illustrate the respective growth of the FSC with the reference map up to a certain limit; the improvement becomes marginal when reducing the overrepresented views further from 2000 to 1000. For the subset with 1000 views, their distribution was near-uniform everywhere except for empty regions (Fig. 2 ▸*f*). Overall, the FSC values with the control 20Ku reconstruction were quite high, but were lower than for the reconstruction with the uniformly distributed 1000 views (Fig. 5 ▸*c*). The reason for this is the presence of empty angular regions, for which our procedure could not recover information, and we considered this result to be the best possible under the given conditions. For these error-free simulated data, the deformation of the 3D reconstruction with the 1Kn subset of projections was rather marginal (Fig. 6 ▸*b*), especially for the regions of interest, corresponding to 0.002 < *p* < 0.004 (Fig. 5 ▸*c*), showing molecular contours and the main features.

This improvement of the results by equalizing the view distribution for error-free data sets by a simple reduction of the number of overrepresented projections is not surprising. A large number of projections is important to filter over errors contained in 2D projections, while this is irrelevant for error-free data. At the same time, information becomes lost when the number of projections falls below a certain limit.

#### 3.1.3. Non-uniformly distributed views with low noise

To analyze the effect of noise, we introduced artificial independent errors into the values of the 2D projections. They were introduced according to the normal law with mean zero and σ equal to 1 r.m.s.d., the value calculated independently for each projection; in the following, we refer to this set of projections as 1σ projections.

We computed the 3D reconstruction with the 10Ku data set of 1σ projections, distributed uniformly, and compared it with the control reconstruction 20Ku. The FSC values were close to those for the error-free data in the medium-resolution interval (Fig. 5 ▸*d*) and slightly lower in higher resolution shells (Fig. 5 ▸*e*). The reconstruction inside the molecular region, corresponding to *p* < 0.004, was as good as for the error-free projections, while it was significantly worse outside (blue curves in Fig. 5 ▸*f*).

For the subset 10Kn of non-uniformly distributed projections with noise, the quality of the 3D reconstruction was similar to that for the error-free set (red curves in Figs. 5 ▸*d* and 5 ▸*e*). The reconstruction with the 1Kn subset of projections, after removing the most overrepresented views, significantly improved the FSC values. Nevertheless, the resulted FSC values were slightly lower than in the similar test with error-free projections, especially for high-resolution shells [compare the continuous and dashed black curves in Figs. 5 ▸(*d*) and 5 ▸(*e*) with the respective red curves]. At the same time, this weaker confidence of the Fourier coefficients did not affect the map accuracy in the molecular region (Fig. 5 ▸*f*). Obviously, both maps were less accurate than the maps obtained with the uniform view distribution, increasing *D* from approximately 0.05 to approximately 0.20. We conclude that for such relatively small errors in the projection values, 1000 projections were sufficient to remove the noise by the respective averaging, and that after removing severely overrepresented views, the residual map distortions were due only to the missed views.

#### 3.1.4. Non-uniformly distributed views with large noise

We then repeated the reconstruction with the uniformly distributed views, but with 3σ errors introduced into the projection values. The FSC values for comparison with the control reconstruction 20Ku were close to those for the error-free data but were significantly worse at higher resolution, starting from approximately 4 Å (compare the red and blue curves in Figs. 5 ▸*g* and 5 ▸*h*).

Fig. 6 ▸(*c*) shows the map reconstructed with 10 000 projections, the same as used for the 10Kn map previously, but this time with 3σ errors introduced into the projection values instead of 1σ errors. The plots in Figs. 5 ▸(*g*) and 5 ▸(*h*) show that the main errors in the recovered Fourier coefficients of medium resolution come mostly from the non-uniform distribution and not from the noise in the data, while for higher resolution it is about half and half. Introducing strong noise into the uniformly distributed set of projections 10Ku does not distort the reconstructed map much inside the molecular region for *p* < 0.005 but does outside it (blue curves in Fig. 5 ▸*i*). The map reconstructed with the non-uniformly distributed set 10Kn reproduces the exact solution as poorly as with the noise-free data set (red curves in Fig. 5 ▸*i*).

For these projections with large noise, in contrast to calculations with the error-free and 1σ noise projections, leaving only 1000 uniformly distributed projections did not improve the FSC at medium resolution (Fig. 5 ▸*g*) and, just the opposite, made it worse at a resolution of approximately 3.5 Å and higher (compare the black and red dashed curves in Fig. 5 ▸*h*). Interestingly, the *D*-function values decrease, indicating some map improvement, even if not as much as for similar calculations with 1σ errors (black and red dashed curves in Figs. 5 ▸*f* and 5 ▸*i*). We hypothesize that this reflects a slight improvement of the Fourier coefficients at medium and low resolution (Fig. 5 ▸*g*) that define the overall shape of the molecular image, even when their confidence is low according to the FSC.

The results of calculations with error-free, 1σ error and 3σ error projections illustrate that in the latter case an insufficiently large number of projections left significant residual errors. This imperfection may be crucial when refining coordinates and *B*-factor values of atomic models versus cryo-EM maps. As a consequence, the simple removal of overrepresented views may not be an optimal protocol.

#### 3.1.5. Reconstructions completing underrepresented views

**Figure 7:**
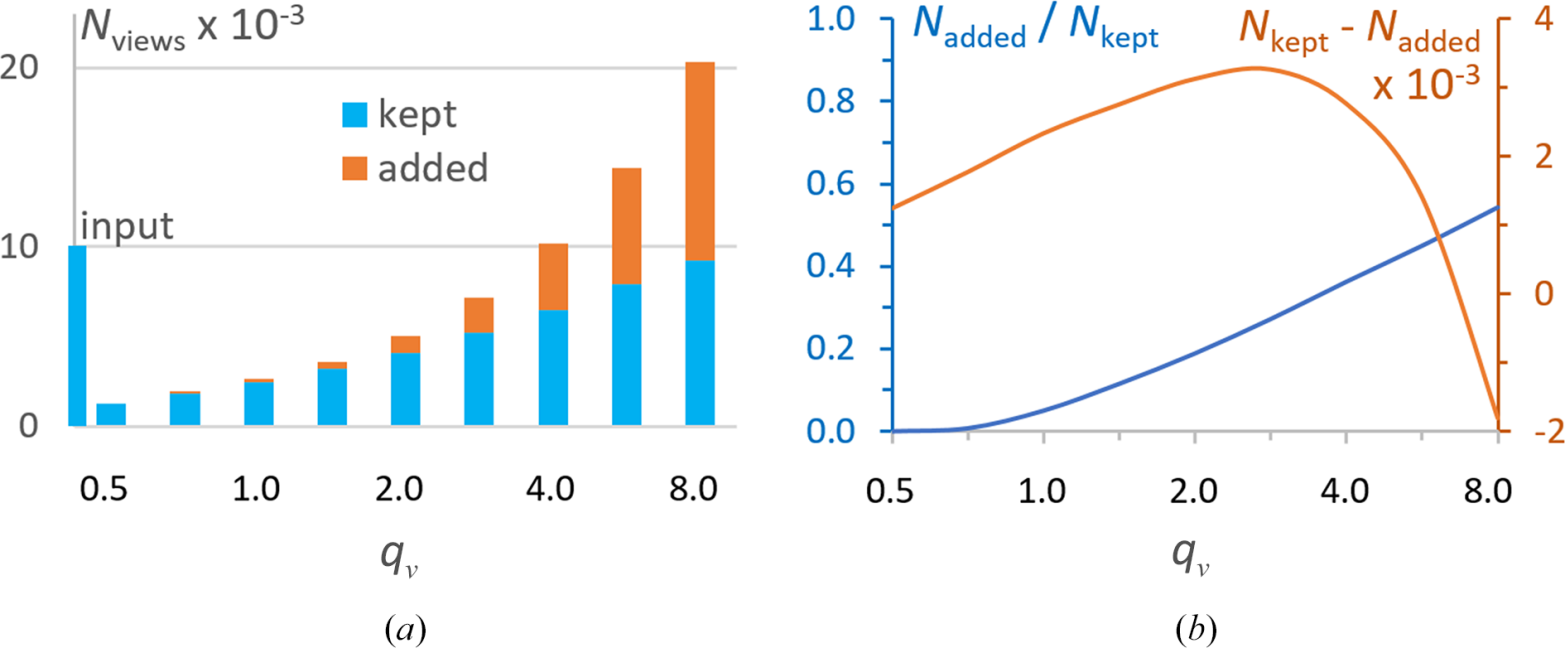
Number of views for the IF2 data as a function of the relative equilibrium frequency. (*a*) Number of removed and added views, in thousands, to reduce the non-uniform distribution. (*b*) Ratio and difference of the added views with respect to the kept views. Both plots are given as a function of the frequency cutoff with respect to the theoretical frequency for the uniform distribution, *q*_ν_ = ν_cut_/ν_uniform_.

Trying to both equilibrate the view distribution and keep the number of views sufficiently large, we combined the removal of overrepresented projections with the introduction of some ‘dummy’ projections to complete underrepresented views, as described in Section 2. Fig. 7 ▸(*a*) shows the number of such views applying different values of the frequency cutoff level *q*_ν_ (relative equilibrium frequency) described in Section 2.2. The ‘dummy’ projections (or, more correctly, extra references to the existing projections) were added with the exact values of the Euler angles describing the direction of the projections, or introducing random perturbations in the Euler angle values, that differed in the different tests, from small mean values up to a relatively huge value of 10° (Figs. 2 ▸*g* and 2 ▸*h*).

The most improved 3D reconstructions were obtained when the total number of views was either the same as before correction, *i.e.* about 10 000, or slightly reduced, up to about 5000. For the given data set, these modified sets were obtained with cutoff parameter values in the range 2 ≤ *q*_ν_ ≤ 4 (Fig. 7 ▸*a*). This corresponds to the highest values of the difference between the number of kept and added ‘dummy’ views (Fig. 7 ▸*b*). While the reconstruction with 10 000 total views gave slightly better FSC values (by 1–2%), the reconstruction with about 5000–6000 total views gave values of the *D* function that were better by 2–3% (curves in magenta in Figs. 5 ▸*g*–5 ▸*i*). Fig. 5 ▸(*i*) also shows that in the region of the molecule, 0 < *p* < 0.005, the reconstruction with such a combined set of projections results in a map of similar quality to the best one that can be obtained using the error-free non-uniformly distributed set, namely the 1Kn set (magenta curve compared with the black continuous curve). Naturally, these estimates are only indicative and can be used for reference; the optimal choice depends on the particular data set of projections and the particular structure.

Introducing artificial deviations in the values of the Euler angles for the generated ‘dummy’ projections made the results marginally worse. We can speculate that the refinement protocol recovered the original Euler angles rather precisely, thus giving the false impression that the distorted projections could better reconstitute the Fourier coefficients corresponding to the missed projections.

### 3.2. Correction of experimental data sets

#### 3.2.1. 70S ribosome from *Staphylococcus aureus*

**Figure 8:**
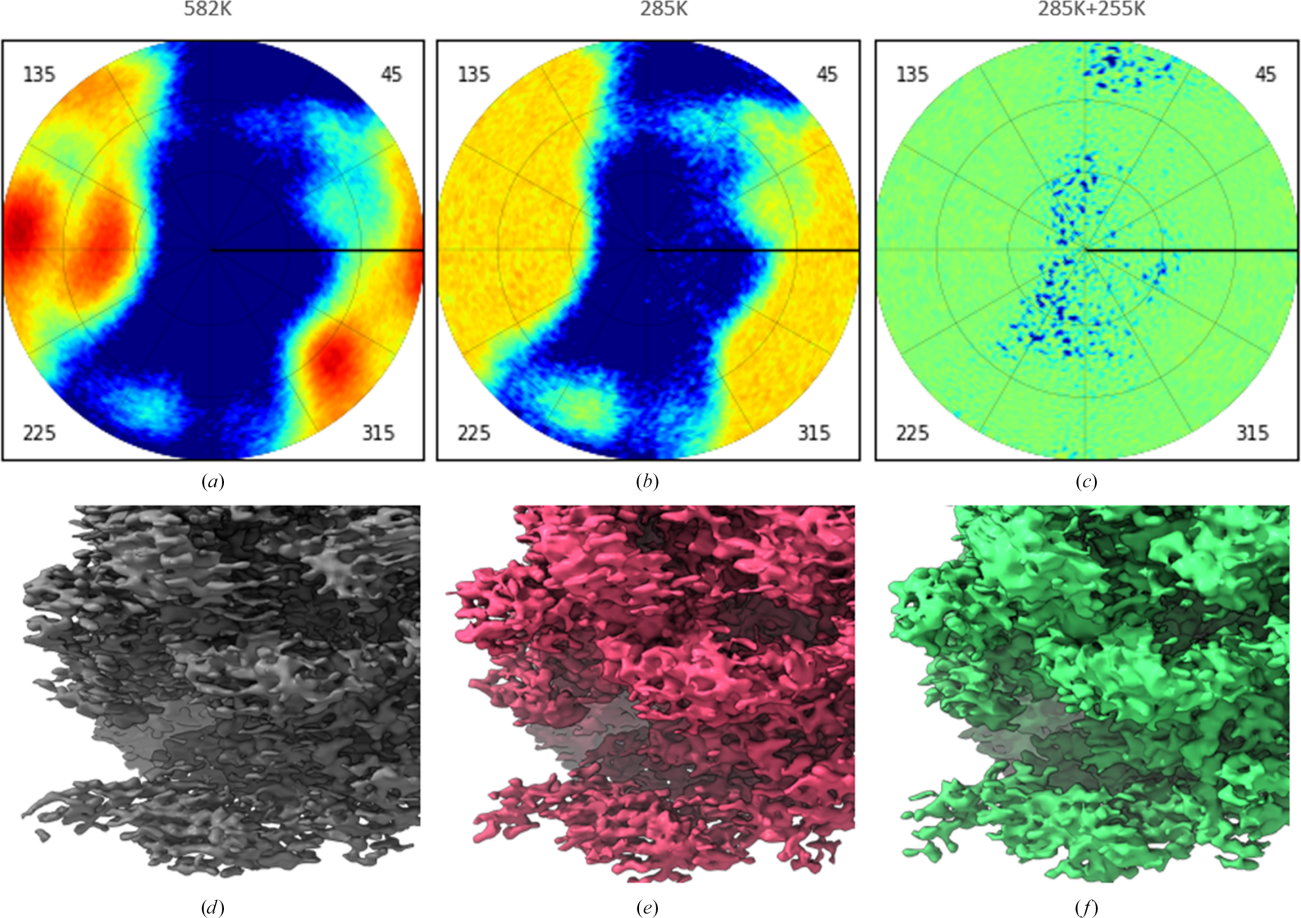
70S *S. aureus* ribosome data sets. (*a*) Initial data set. (*b*) Data set after removing half of the most overrepresented views. (*c*) Data set after removing half of the most overrepresented views and completing with approximately the same number of underrepresented views. Top row: view distribution. The color scheme for the diagrams is the same as in Fig. 1 ▸. Bottom row: maps of the respective 3D reconstructions. The maps were plotted with *Chimera* (Pettersen *et al.*, 2004 ▸).

The initial 3D reconstruction for the 70S *S. aureus* ribosome data set was performed with all approximately 582 000 cryo-EM projections in the presence of three clusters of overrepresented views (Fig. 8 ▸*a*). The map corresponding to the 3D reconstruction with this data set shows some anisotropy (Fig. 8 ▸*d*). Removing about half of the most overrepresented views (about 285 000 projections left) blurred these three clusters of the projections (Fig. 8 ▸*b*). This made the respective map more homogeneous (compare Fig. 8 ▸*a* with Fig. 8 ▸*b*), while, as expected, the overall resolution slightly decreased. Nevertheless, the map was still distorted in some directions, as previously (Fig. 8 ▸*e*).

Finally, we removed the same number of overrepresented projections and completed the underrepresented projections by repetitive references to them, as explained in Section 2, making the total number of views equal to ∼551 000. This time the angular space was covered nearly uniformly (Fig. 8 ▸*c*). This was possible because initially the set of views contained those for most directions, but with very different frequencies. Two opposite effects could be observed. On one hand, according to the software *RELION*, the nominal overall resolution decreased from 3.0 Å for the initial map to 3.1 Å for the intermediate map and to 3.2 Å for the last map. On the other hand, this time the map was fully equilibrated, with no trace of the previous deformations or stripes in the densities (Fig. 8 ▸*f*) and therefore was easier to interpret.

**Figure 9:**
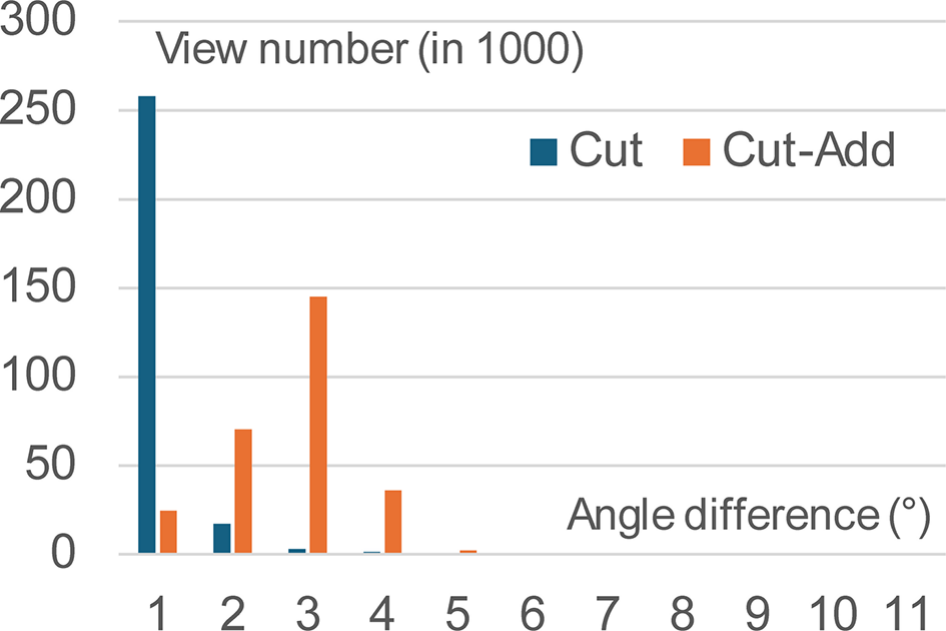
Histogram of the angular difference between the original and modified set of views. The two sets are obtained by removing overrepresented views (Cut) and by additionally completing the underrepresented views (Cut-Add).

To analyze the principal source of the observed improvement in the 3D reconstruction, we evaluated how much the projection directions changed during further refinement in reconstructions obtained with the ‘reduced’ and the ‘reduced plus completed’ data sets, in comparison with the original 3D reconstruction. Fig. 9 ▸ shows the distribution of angular differences between the corresponding views. For the reconstruction based on the data set in which projections corresponding to underrepresented views were included in multiple copies, the directions of all copies of a given view were identical; therefore, the angular difference with respect to the original direction was counted only once for each such view. The mean angular deviation was 1.09° for the reconstruction using the reduced set of views and 2.66° for the reconstruction using the completed set obtained after removing overrepresented views. This indicates that angular refinement is now allowed while the angular spread is rather small.

At the same time, for both modified data sets, that with overrepresented views removed and that with underrepresented views completed, approximately 3300 views (about 1% of the total number of the views compared in each case) changed their assigned directions by more than 10°, with some deviations reaching up to 85° (not visible in Fig. 9 ▸, as these correspond to fewer than 100 views per bin). This suggests that equalizing the set of projections in this manner may facilitate the iterative improvement of view directions by improving the quality of the 3D reconstruction because correction allows the exploration of new angles as the refinement is no longer biased by a ‘stripy’ reference.

In an extra test, we introduced artificial perturbations into the directions of the added copies of underrepresented views, intended to enhance the exploration of Fourier space. The 3D reconstruction procedure refined these views back to their original directions, even when the mean introduced error was as large as 10° (data not shown).

#### 3.2.2. 80S human ribosome data

**Figure 10:**
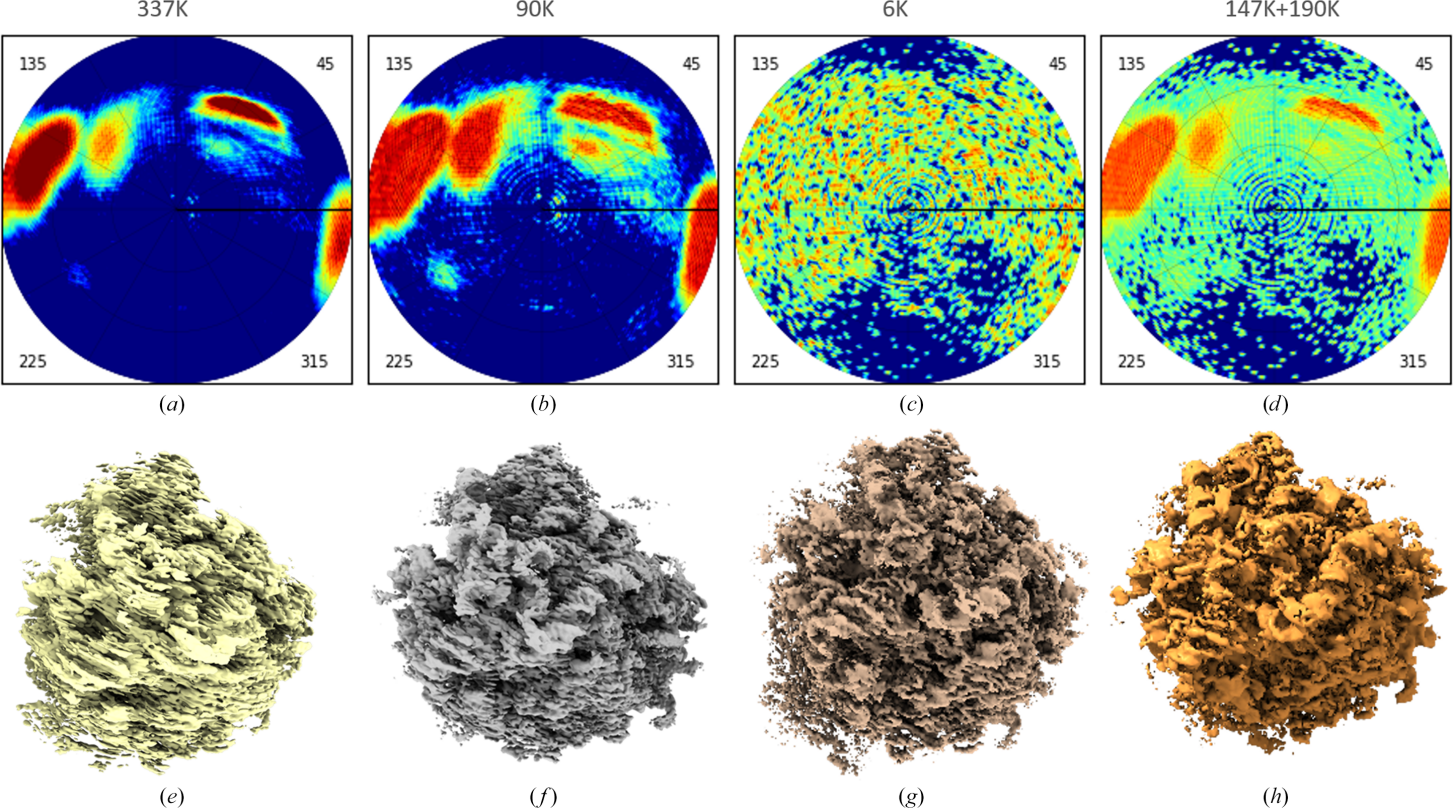
Human 80S ribosome data. Top row, view distribution; bottom row, the respective maps. (*a*, *e*) Initial data set, about 337 000 projections; the map shows characteristic stripes and distortions. (*b*, *f*) Most overrepresented projections removed; about 90 000 left. (*c*, *g*) The same; about 6000 left. (*d*, *h*) The set composed of about 337 000 projections after removal of about half of the most overrepresented projections and completing with a similar number of underrepresented projections. The color scheme for the diagrams is the same as in Fig. 1 ▸. Maps were plotted with *Chimera* (Pettersen *et al.*, 2004 ▸).

As shown in Section 2.3, a non-uniform distribution of views for the human 80S ribosome (Fig. 10 ▸*a*) leads to the 3D reconstruction being blurred anisotropically (Fig. 10 ▸*e*). Trying to improve the map, we first reduced the overrepresented views, randomly removing their extra copies. This left us with about 90 000 views. Their distribution was still significantly non-uniform (Fig. 10 ▸*b*) and the respective 3D reconstruction showed the same kind of defects as before (Fig. 10 ▸*f*).

Fig. 10 ▸(*c*) shows the view distribution when we tried to equalize the distribution further by removing the most overrepresented views. The corresponding set was composed of only about 6500 views. While the corresponding 3D reconstruction was not blurred (Fig. 10 ▸*g*), the resolution was significantly reduced (*RELION* provided a value of 4.8 Å compared with 4.0 Å for the initial map).

Finally, we removed about half of the most overrepresented views and added a similar number of ‘dummy’ views corresponding to underrepresented projections, taking each of them in several copies, according to their frequency. As discussed previously, this distribution was a compromise between the number of experimental projections kept and an attempt to make the view distribution more uniform (Fig. 10 ▸*d*). This obtained 3D reconstruction was not deformed at all and contained more details (the *RELION* resolution estimate was 4.0 Å) in comparison with the previous reconstruction and became much clearer to interpret (Fig. 10 ▸*h*).

## 4. Discussion

By their definition, irremovable errors both in crystallography and in cryo-EM cannot be corrected by any kind of refinement procedure and require special treatment. For example, in crystallography, the missing part of the atomic model can be modeled either theoretically (Lunin & Skovoroda, 1995 ▸; Bricogne & Irwin, 1996 ▸; Pannu & Read, 1996 ▸; Murshudov *et al.*, 1997 ▸; Lunin *et al.*, 2002 ▸) or explicitly by introducing dummy atoms (Isaacs & Agarwal, 1977 ▸; Lunin & Urzhumtsev, 1984 ▸; Lamzin & Wilson, 1993 ▸). Some methodologically similar approaches may be tried to correct irremovable errors in cryo-EM; severely non-uniformly distributed views are an example of such errors where the explicit introduction of ‘dummy views’ can be tried.

The results of 3D reconstruction depend both on the available set of 2D projections and on the reconstruction procedure. We tested the most straightforward procedure using the software *RELION*, varying the number of references to a given set of the 2D projections from zero, *i.e.* removing a reference to the respective projection, to several, *i.e.* artificially considering multiple copies of the same projection, with or without perturbating its view parameters, during reconstruction. Naturally, this can be seen as explicit weighting of such projections.

We observed that, as expected, for projections with small errors in their values, the simple removal of the overrepresented views can help to some extent, unless the number of the projections left becomes excessively small. On the contrary, for data with significant errors, the simple removal of overrepresented projections is counterproductive. Removing the most overrepresented views and repeating information for underrepresented projections, thus making the final distribution almost uniformly distributed, results in 3D reconstructions with less deformation. Our tests suggest that such formal explicit correction of the set of projections should keep the total number of references close enough to that in the original set.

As our example with the 40S small ribosomal subunit shows, a non-uniform distribution of views does not necessarily result in strong map deformation. Obviously, the impact of overrepresented and underrepresented views depends on the extent of the non-uniformity, which can be expressed quantitatively (Naydenova & Russo, 2017 ▸; Baldwin & Lyumkis, 2021 ▸; Urzhumtsev, 2026 ▸). Conversely, non-uniformity in the distribution of views is far from being the sole source of map deformation. Nevertheless, correcting errors arising from this imperfection should help other image-processing procedures to be performed more successfully, in particular allowing the exploration of other angular assignments as the refinement is no longer biased by a view-distorted reference because the 3D reconstruction provides a better internal reference during cryo-EM structure refinement.

While the map improvement after completing the underrepresented views with extra copies is not always spectacular, new maps can facilitate model building when used alone or in parallel with the initial maps. Also, they may serve as a better reference map for further refinement of the 3D reconstructions, as well as for real-space refinement of atomic models.

To analyze the results of the reconstruction, we used traditional measures such as the FSC function with respect to the reference reconstruction. We observed that this information is not sufficient to reflect the actual similarity of maps and especially their appearance during visual analysis. For such goals, we use the discrepancy function *D* (Lunin, 1988 ▸), introduced earlier in crystallography. We observed examples when some decrease in the FSC values calculated from the half-maps in fact corresponded to a map improvement inside the molecular region. We also noted that FSC curves calculated and shown on a uniform scale in Å^−3^ are more relevant statistically and have some advantages when enlarged at higher resolution intervals.

View distributions were analyzed with the program *VUE* (Urzhumtseva *et al.*, 2024 ▸), which now also allows routine modification of lists of 2D projections, making the view distribution more uniform. Another advantage is that this program illustrates these distributions ‘on-the-fly’. The program is available by request from one of the authors (AU) or from the website https://git.cbi.igbmc.fr/sacha/vue-cryo-em-software. 

## References

[bb1] Afonine, P. V., Klaholz, B. P., Moriarty, N. W., Poon, B. K., Sobolev, O. V., Terwilliger, T. C., Adams, P. D. & Urzhumtsev, A. (2018). *Acta Cryst.* D**74**, 814–840. 10.1107/S2059798318009324 PMC6130467 30198894

[bb2] Baldwin, P. R., Aiyer, S., Strutzenberg, T. S. & Lyumkis, D. (2023). *Microsc. Microanal.* **29**, 1021–1023.

[bb3] Baldwin, P. R. & Lyumkis, D. (2021). *Prog. Biophys. Mol. Biol.* **160**, 53–65. 10.1016/j.pbiomolbio.2020.06.003 PMC7785567 32645314

[bb4] Boisset, N., Penczek, P., Taveau, J. C., You, V., de Haas, F. & Lamy, J. (1998). *Ultramicroscopy*, **74**, 201–207.

[bb5] Bricogne, G. & Irwin, J. (1996). *Proceedings of the CCP4 Study Weekend. Macromolecular Refinement*, edited by E. J. Dodson, M. Moore, A. Ralph & S. Bailey, pp. 85–92. Warrington: Daresbury Laboratory.

[bb6] Diamond, R. (1971). *Acta Cryst.* A**27**, 436–452.

[bb7] Fokine, A. & Urzhumtsev, A. (2002). *Acta Cryst.* D**58**, 1387–1392. 10.1107/S0907444902010284 12198293

[bb8] Grant, T., Rohou, A. & Grigorieff, N. (2018). *eLife*, **7**, e35383. 10.7554/eLife.35383 PMC5854467 29513216

[bb9] Harauz, G. & van Heel, M. (1986). *Optik*, **73**, 146–156.

[bb10] Heymann, J. B., Chagoyen, M. & Belnap, D. M. (2005). *J. Struct. Biol.* **151**, 196–207. 10.1016/j.jsb.2005.06.001 16043364

[bb11] Holvec, S., Barchet, C., Lechner, A., FréChin, L., De Silva, S. N. T., Hazemann, I., Wolff, P., von Loeffelholz, O. & Klaholz, B. P. (2024). *Nat. Struct. Mol. Biol.* **31**, 1251–1264. 10.1038/s41594-024-01274-x 38844527

[bb12] Isaacs, N. W. & Agarwal, R. C. (1977). *Proc. Natl Acad. Sci. USA*, **74**, 2835–2839. 10.1073/pnas.74.7.2835 PMC431311 268634

[bb13] Khatter, H., Myasnikov, A. G., Natchiar, S. K. & Klaholz, B. P. (2015). *Nature*, **520**, 640–645. 10.1038/nature14427 25901680

[bb14] Kühlbrandt, W. (2014). *Science*, **343**, 1443–1444. 10.1126/science.1251652 24675944

[bb15] Lamzin, V. S. & Wilson, K. S. (1993). *Acta Cryst.* D**49**, 129–147. 10.1107/S0907444992008886 15299554

[bb16] Liebschner, D., Afonine, P. V., Baker, M. L., Bunkóczi, G., Chen, V. B., Croll, T. I., Hintze, B., Hung, L.-W., Jain, S., McCoy, A. J., Moriarty, N. W., Oeffner, R. D., Poon, B. K., Prisant, M. G., Read, R. J., Richardson, J. S., Richardson, D. C., Sammito, M. D., Sobolev, O. V., Stockwell, D. H., Terwilliger, T. C., Urzhumtsev, A. G., Videau, L. L., Williams, C. J. & Adams, P. D. (2019). *Acta Cryst.* D**75**, 861–877. 10.1107/S2059798319011471 PMC6778852 31588918

[bb17] Lunin, V. Yu. (1988). *Acta Cryst.* A**44**, 144–150.

[bb18] Lunin, V. Y., Afonine, P. V. & Urzhumtsev, A. G. (2002). *Acta Cryst.* A**58**, 270–282. 10.1107/s0108767302001046 11961289

[bb19] Lunin, V. Yu. & Skovoroda, T. P. (1995). *Acta Cryst.* A**51**, 880–887.

[bb20] Lunin, V. Yu. & Urzhumtsev, A. G. (1984). *Acta Cryst.* A**40**, 269–277.

[bb21] Murshudov, G. N., Vagin, A. A. & Dodson, E. J. (1997). *Acta Cryst.* D**53**, 240–255. 10.1107/S0907444996012255 15299926

[bb22] Natchiar, S. K., Myasnikov, A. G., Kratzat, H., Hazemann, I. & Klaholz, B. P. (2017). *Nature*, **551**, 472–477. 10.1038/nature24482 29143818

[bb23] Naydenova, K. & Russo, C. J. (2017). *Nat. Commun.* **8**, 62. 10.1038/s41467-017-00782-3 PMC5607000 28931821

[bb24] Orlov, I. M., Morgan, D. G. & Cheng, R. H. (2006). *J. Struct. Biol.* **154**, 287–296. 10.1016/j.jsb.2006.03.007 16690323

[bb25] Orlov, I. M., Rochel, N., Moras, D. & Klaholz, B. P. (2011). *EMBO J.* **31**, 291–300. 10.1038/emboj.2011.445 PMC3261568 22179700

[bb26] Pannu, N. S. & Read, R. J. (1996). *Proceedings of the CCP4 Study Weekend. Macromolecular Refinement*, edited by E. J. Dodson, M. Moore, A. Ralph & S. Bailey, pp. 75–84. Warrington: Daresbury Laboratory.

[bb27] Peng, L.-M. (1999). *Micron*, **30**, 625–648.

[bb28] Pettersen, E. F., Goddard, T. D., Huang, C. C., Couch, G. S., Greenblatt, D. M., Meng, E. C. & Ferrin, T. E. (2004). *J. Comput. Chem.* **25**, 1605–1612. 10.1002/jcc.20084 15264254

[bb29] Punjani, A., Rubinstein, J. L., Fleet, D. J. & Brubaker, M. A. (2017). *Nat. Methods*, **14**, 290–296. 10.1038/nmeth.4169 28165473

[bb30] Saxton, W. O. & Baumeister, W. (1982). *J. Microsc.* **127**, 127–138. 10.1111/j.1365-2818.1982.tb00405.x 7120365

[bb31] Scheres, S. H. W. (2012). *J. Struct. Biol.* **180**, 519–530. 10.1016/j.jsb.2012.09.006 PMC3690530 23000701

[bb33] Shaikh, T. R., Gao, H., Baxter, W. T., Asturias, F. J., Boisset, N., Leith, A. & Frank, J. (2008). *Nat. Protoc.* **3**, 1941–1974. 10.1038/nprot.2008.156 PMC2737740 19180078

[bb34] Simonetti, A., Marzi, S., Fabbretti, A., Hazemann, I., Jenner, L., Urzhumtsev, A., Gualerzi, C. O. & Klaholz, B. P. (2013). *Acta Cryst.* D**69**, 925–933. 10.1107/S0907444913006422 PMC3663118 23695237

[bb35] Sorzano, C. O. S., Semchonok, D., Lin, S.-C., Lo, Y.-C., Vilas, J. L., Jiménez-Moreno, A., Gragera, M., Vacca, S., Maluenda, D., Martínez, M., Ramírez-Aportela, E., Melero, R., Cuervo, A., Conesa, J. J., Conesa, P., Losana, P., Caño, L. D., de la Morena, J. J., Fonseca, Y. C., Sánchez-García, R., Strelak, D., Fernández-Giménez, E., de Isidro, F., Herreros, D., Kastritis, P. L., Marabini, R., Bruce, B. D. & Carazo, J. M. (2021). *J. Struct. Biol.* **213**, 107695. 10.1016/j.jsb.2020.107695 33421545

[bb36] Stagg, S. M., Noble, A. J., Spilman, M. & Chapman, M. S. (2014). *J. Struct. Biol.* **185**, 418–426. 10.1016/j.jsb.2013.12.010 PMC4001718 24384117

[bb37] Unger, V. M. (2000). *Acta Cryst.* D**56**, 1259–1269. 10.1107/s0907444900011252 10998622

[bb38] Urzhumtsev, A. G. (2025). *Acta Cryst.* D**81**, 621–629. 10.1107/S2059798325008526 PMC12576846 41059947

[bb39] Urzhumtsev, A. G. (2026). *Acta Cryst.* D**82**, 100–112. 10.1107/S2059798325011611 41553141

[bb40] Urzhumtsev, A., Afonine, P. V., Lunin, V. Y., Terwilliger, T. C. & Adams, P. D. (2014). *Acta Cryst.* D**70**, 2593–2606. 10.1107/S1399004714016289 PMC4188004 25286844

[bb41] Urzhumtsev, A. & Lunin, V. Y. (2022). *IUCrJ*, **9**, 728–734. 10.1107/S2052252522008260 PMC9634607 36381145

[bb42] Urzhumtsev, A. G., Urzhumtseva, L. M. & Lunin, V. Y. (2022). *Acta Cryst.* D**78**, 1451–1468. 10.1107/S2059798322010907 36458616

[bb43] Urzhumtseva, L., Barchet, C., Klaholz, B. P. & Urzhumtsev, A. G. (2024). *J. Appl. Cryst.* **57**, 865–876. 10.1107/S1600576724002383 PMC11151668 38846771

[bb44] van Heel, M., Keegstra, W., Schutter, W. & van Bruggen, E. F. J. (1982). *The Structure and Function of Invertebrate Respiratory Proteins: EMBO Workshop, Leeds, UK, Life Chemistry Reports Supplement 1*, edited by E. J. Wood. pp. 69–73. Harwood Academic Publishers.

